# Bridging the Gap in Lung Cancer: Novel Biomarkers, Metabolic Drivers, and Advanced Therapeutics from Bench to Bedside

**DOI:** 10.3390/biomedicines14081681

**Published:** 2026-07-27

**Authors:** Jeongmi Lee, Seung Hyeun Lee

**Affiliations:** 1 Division of Pulmonary, Allergy, and Critical Care Medicine, Department of Internal Medicine, College of Medicine, Kyung Hee University, Seoul 02447, Republic of Korea; wjdal0916@naver.com; 2 Department of Precision Medicine, Graduate School, Kyung Hee University, Seoul 02447, Republic of Korea

The therapeutic paradigms for both non-small cell lung cancer (NSCLC) and small cell lung cancer (SCLC) have entered an unprecedented era of precision oncology, a transformative evolution fundamentally driven by the seamless integration of translational science [1]. Reflecting on this dynamic clinical and laboratory landscape, this Special Issue, titled “Advances in Lung Cancer: From Bench to Bedside,” compiles a curated collection of nine high-impact articles (eight original research papers and one comprehensive literature review). Together, these contributions epitomize the essential bidirectional pipeline of modern thoracic oncology: bridging sophisticated laboratory discoveries (“the bench”), such as novel proteomic profiles [2] and metabolic vulnerabilities [3], directly into the refinement of real-world therapeutic strategies (“the bedside”), including advanced radiotherapy planning [4], real-world evidence of immune checkpoint inhibition [5], and mRNA vaccines as a modern immunotherapy platform [6]. By dismantling the traditional silos between basic molecular biology and clinical practice, the diverse research presented herein not only unravels the complex mechanisms of therapeutic resistance and tumor progression but also establishes pragmatic, non-invasive diagnostic and monitoring frameworks that promise to optimize treatment sequencing and ultimately advance long-term patient survivorship.

The therapeutic landscape for advanced lung adenocarcinoma has been deeply transformed, as evidenced by the clinical success of epidermal growth factor receptor tyrosine kinase inhibitors (EGFR-TKIs) [7]. Although EGFR-TKIs initially yield remarkable objective response rates in patients with sensitizing EGFR mutations, the inevitable emergence of acquired resistance remains a major clinical barrier that limits long-term survival. Although genomic alterations such as secondary T790M mutation or bypass pathway activation are well-established mechanisms of relapse, they do not fully account for all clinical resistance phenotypes [8]. Consequently, simple blood-based biomarkers capable of predicting early progression and decoding the heterogeneous molecular microenvironment of TKI-resistant lung adenocarcinoma (LUAD) need to be urgently identified.

In the first research article of this issue, Raungrut et al. addressed this critical clinical challenge through a serum proteomic profiling study aimed at identifying predictive biomarkers of relapse in patients with advanced LUAD receiving first- or second-generation EGFR-TKI therapy [2]. Using high-throughput liquid chromatography-mass spectrometry in independent discovery and validation cohorts, the authors successfully revealed distinct protein expression profiles that distinguished early relapsing patients from long-term responders. Mechanistically, this study highlighted the translocated promoter region (TPR) and fibrinogen alpha chain (FGA) as pivotal serum biomarkers. The authors demonstrated that elevated baseline or pre-relapse levels of these proteins strongly correlate with shortened progression-free survival (PFS). The findings suggest that the TPR and FGA levels may be potential predictors of relapse after EGFR-TKI treatment. By establishing TPR and FGA as viable liquid biopsy-based predictive biomarkers, this study offers thoracic oncologists a potentially noninvasive, blood-based tool for identifying patients at high risk of early treatment failure before radiographic evidence of disease progression emerges. Clinically, anticipating resistance via serum proteomic shifts could facilitate a timely transition to third-generation TKIs or prompt the early initiation of rational combination regimens. Furthermore, integrating these circulating proteomic signatures with routine genomic testing may optimize therapeutic sequencing, mitigate the burden of acquired resistance, and ultimately improve long-term survival in patients with advanced lung adenocarcinoma.

First-line pembrolizumab monotherapy has been the standard of care for metastatic non-small cell lung cancer (NSCLC) with a programmed death-ligand 1 (PD-L1) tumor proportion score (TPS) ≥ 50% since the pivotal KEYNOTE-024 trial, whose 5-year follow-up reported a median overall survival (OS) of 26.3 months [9]. However, the highly selected patient population enrolled in registration trials may not fully reflect routine clinical practice, and real-world evidence from Eastern Europe—a region with one of the highest lung cancer burdens worldwide—remains limited.

In the second research article, Marković et al. addressed this knowledge gap with a retrospective, multicenter analysis of 225 patients treated at the two largest lung cancer centers in Serbia [5]. After a median follow-up of 18.1 months, the median PFS and OS were 9.7 and 17.0 months, respectively, whereas the overall response and disease control rates were 36.4% and 73.4%, respectively. Although these outcomes were inferior to those reported in KEYNOTE-024, they were consistent with findings from other real-world registries [10,11,12]. The authors reasonably attribute this difference to the inclusion of patients with poor performance status, with Eastern Cooperative Oncology Group Performance Status (ECOG PS) ≥ 2 accounting for 18.7% of the cohort, a group typically excluded from clinical trials. Multivariable analysis identified three independent predictors of both longer PFS and OS: good performance status (ECOG PS 0–1), very high PD-L1 expression (TPS ≥ 90%), and the occurrence of immune-related adverse events (irAEs). Notably, ECOG PS ≥ 2 was associated with an OS hazard ratio > 5, whereas irAEs—predominantly low-grade cutaneous, thyroid, and gastrointestinal events—were associated with improved clinical outcomes, consistent with the surrogate-of-activity hypothesis from Cortellini et al.’s large real-world cohort [13]. Collectively, these findings suggest that three readily available variables stratify the benefits of first-line pembrolizumab treatment. The performance status signal reinforces the observation that patients with frailty generally experience poorer outcomes, although the IPSOS trial demonstrated that immune checkpoint inhibitor (ICI) monotherapy may still outperform single-agent chemotherapy in patients who are ineligible for platinum-based treatment [14]. Furthermore, the association between TPS ≥ 90% and improved outcomes strengthens the growing rationale for moving beyond the binary 50% threshold toward more refined PD-L1 stratification when selecting between monotherapy and combination regimens. Although the irAE association is biologically plausible, it warrants caution given the immortal-time bias of on-treatment events, the limitations of dichotomized analyses, and a modest sample size. Prospective designs that use continuous biomarker modeling and time-dependent analyses are required to translate consistent observations into actionable tools. Overall, these findings validate established prognostic factors in a real-world Eastern European cohort and may support more personalized treatment approaches in patients receiving pembrolizumab monotherapy.

Stereotactic body radiation therapy (SBRT) has firmly established itself as the guideline-recommended standard of care in patients with inoperable early-stage NSCLC [15]. Conventionally, pathological confirmation via biopsy is a prerequisite for initiating definitive local treatment. However, in clinical practice, a significant subset of older patients or individuals with severe cardiopulmonary comorbidities face prohibitive procedural risks from invasive tissue biopsies or present with anatomically inaccessible nodules. In these challenging scenarios, managing solitary or synchronous multiple pulmonary nodules based solely on high-suspicion computed tomography (CT) and positron emission tomography (PET)/CT imaging poses a crucial clinical dilemma, requiring a careful balance between the need for early intervention and the risks of empirical noninvasive management.

In the third research article, Csiki et al. provided important clinical support for this empirical paradigm through a comprehensive retrospective cohort study evaluating the effectiveness and safety of SBRT for solitary or synchronous multiple pulmonary nodules highly suspicious for early-stage lung cancer without pathological confirmation [4]. Analyzing long-term follow-up data, the authors demonstrate remarkable therapeutic efficacy; One- and two-year local control rates were 89.8% and 94.3%, respectively, with a 51.4% complete response rate at two years. Mean overall survival was 49.6 months. No grade ≥ 3 toxicities were observed. The data demonstrate that empirical SBRT for synchronous multiple nodules is both feasible and highly effective, maintaining robust local control without inducing excessive, overlapping radiation pneumonitis or severe treatment-related toxicities. These findings carry practical weight for the growing population of elderly, comorbid patients in whom tissue confirmation is unsafe or unattainable. The authors reinforce that rigorous multidisciplinary, PET/CT–based selection can substitute for histology without sacrificing curative intent [4]. The results support the matched-cohort evidence showing no meaningful difference in local control or survival between clinically and pathologically diagnosed lesions [16], underscoring that withholding definitive treatment while awaiting unobtainable pathology carries a greater risk.

SCLC is an exceptionally aggressive neuroendocrine malignancy characterized by a rapid doubling time, early widespread metastasis, and a dismal 5-year survival rate [17]. Despite the initial and often dramatic responses to first-line platinum-based chemotherapy combined with ICIs, most patients experience relapse [18]. Although genomic profiling has extensively documented the universal loss of tumor suppressors *RB1* and *TP53*, actionable oncogenic drivers in SCLC remain critically sparse [19]. Consequently, alterations in tumor metabolism, specifically amino acid metabolic reprogramming, have emerged as a compelling therapeutic frontier for identifying novel metabolic vulnerabilities and overcoming chemoresistance in this challenging disease.

In another study, Jeong et al. identified a pivotal metabolic dependency in SCLC by demonstrating that asparagine synthetase (ASNS) promotes tumor growth and survival in SCLC cells [3]. Using comprehensive in vitro and in vivo models, the authors showed that ASNS expression was markedly elevated in SCLC tissues compared with normal lung tissue. Mechanistically, genetic silencing or pharmacological inhibition of ASNS severely impairs cell proliferation, suppresses clonogenic potential, and induces apoptotic cascades under nutrient-deprived conditions. These findings demonstrate that asparagine synthetase contributes to tumor growth and metabolic adaptability in SCLC. Collectively, these results support the functional role of asparagine synthetase in malignant progression and suggest that targeting asparagine metabolism may represent a potential therapeutic approach for this aggressive subtype of lung cancer. By demonstrating that ASNS is selectively overexpressed in SCLC and that its depletion suppresses proliferation, clonogenicity, and xenograft growth, the authors identified asparagine metabolism as a therapeutic vulnerability. In addition, ASNS emerged as a candidate biomarker, with amplification associated with shorter overall survival. Translational appeal is heightened by l-asparaginase, an agent already in clinical use that recapitulates ASNS loss in solid-tumor models and offers a near-term combination strategy [20]. Nevertheless, two important limitations warrant consideration. First, the evidence remains largely preclinical, relying on cell lines, murine models, and public datasets; therefore, the efficacy and therapeutic window in patients remain unproven. Second, transporter-mediated asparagine uptake may blunt single-agent ASNS inhibition, arguing for combinatorial approaches and ASNS-stratified patient selection. Prospective validation in patient-derived tumors, alongside biomarker-guided trials that pair ASNS blockade with asparaginase or mTOR inhibition, is needed before these findings can reshape SCLC management.

The therapeutic paradigm for NSCLC has been revolutionized by the clinical success of ICIs targeting either PD-1/PD-L1 or CTLA-4 pathways. Despite these advances, primary and acquired resistance, along with low tumor mutational burden or cold tumor immune microenvironments in a subset of patients, continue to limit the durability of the response and long-term survival [21]. To overcome these barriers, numerous studies have pursued novel strategies to prime and robustly direct the host immune system against tumor-specific antigens. In this clinical landscape, mRNA vaccine technology, leveraging unprecedented rapid manufacturing scalability and precise antigen-encoding capabilities validated during the coronavirus disease 2019 pandemic, has emerged as a highly promising and versatile platform for expanding the horizons of modern lung cancer immunotherapy [22].

In the fifth article, Kabut et al. presented a comprehensive and timely literature review that delineated the current clinical state, underlying immunological mechanisms, and ongoing human trials of mRNA vaccines specifically tailored for NSCLC [6]. The authors categorized these vaccines into tumor-associated antigen-based options, which target shared, overexpressed proteins, and personalized neoantigen-based vaccines, which are designed according to patient-specific tumor mutations. This study highlights advanced lipid nanoparticle delivery platforms that protect mRNA transcripts and ensure their efficient uptake by antigen-presenting cells, thereby driving robust CD4+ and CD8+ T cell-mediated antitumor responses. Furthermore, the authors synthesized critical data demonstrating that while single-agent efficacy remains modest, combining mRNA vaccines with traditional systemic ICIs yields profound synergistic antitumor activity by simultaneously driving de novo T-cell infiltration and preventing subsequent adaptive immune exhaustion. From a translational and clinical standpoint, this exhaustive review serves as a crucial roadmap for integrating therapeutic vaccines into routine thoracic oncology practice. The true clinical value of mRNA vaccines lies in their potential to transform immunologically “cold” tumors into “hot” ones, thereby potentially sensitizing non-responders to checkpoint blockade or preventing recurrence in the adjuvant setting [23]. Nevertheless, as the authors rightly emphasize, several clinical hurdles must be addressed before widespread adoption, including the optimization of neoantigen prediction algorithms, identification of definitive predictive biomarkers to select ideal candidates, and mitigation of potential overlapping irAEs in combination regimens. Looking ahead, this review emphasizes that the precise coordination of antigen vaccination and immune checkpoint inhibition represents one of the most logically sound and clinically impactful strategies for achieving durable curative responses in patients with advanced NSCLC.

In conclusion, each of the five investigations detailed in this Special Issue offers a distinct yet profoundly interconnected contribution to the collective fight against thoracic malignancies. By stimulating interdisciplinary dialogue and inspiring novel translational hypotheses, we trust that the insights shared within “Advances in Lung Cancer: From Bench to Bedside” will ultimately empower the global oncology community to dismantle therapeutic resistance, refine personalized sequencing strategies, and significantly improve long-term survival outcomes for patients with lung cancer.

## Abbreviations

The following abbreviations are used in this manuscript:

NSCLC	Non-small cell lung cancer
SCLC	Small cell lung cancer
EGFR-TKIs	Epidermal growth factor receptor tyrosine kinase inhibitors
LUAD	Lung adenocarcinoma
TPR	Translocated promoter region
FGA	Fibrinogen alpha chain
PFS	Progression-free survival
PD-L1	Programmed death-ligand 1
TPS	Tumor proportion score
OS	Overall survival
ECOG PS	Eastern Cooperative Oncology Group Performance Status
ICI	Immune checkpoint inhibitor
SBRT	Stereotactic body radiation therapy
ASNS	Asparagine synthetase
irAE	Immune-related adverse event

## References

[1] Hendriks L.E.L., Remon J., Faivre-Finn C., Garassino M.C., Heymach J.V., Kerr K.M., Tan D.S.W., Veronesi G., Reck M. (2024). Non-small-cell lung cancer. Nat. Rev. Dis. Primers.

[2] Raungrut P., Chiangjong W., Masjon T., Maungchanburi S., Ruklert T., Nakwan N. (2025). Proteomic Profiling Reveals TPR and FGA as Predictive Serum Biomarkers of Relapse to First- and Second-Generation EGFR-TKIs in Advanced Lung Adenocarcinoma. Biomedicines.

[3] Jeong M., Kim B.C., Choi H.J., Lee G.T., Jang S.M., Kim K.B. (2025). Asparagine synthetase (ASNS) Drives Tumorigenicity in Small Cell Lung Cancer. Biomedicines.

[4] Csiki E., Bolcskei D., Barabas M., Gal K., Mikaczo J., Miklos S., Trasi K., Solymosi D., Papp J., Simon M. (2025). The Effectiveness of SBRT for Solitary or Synchronous Multiple Pulmonary Nodules Suspicious of Early-Stage Lung Cancer Without Pathological Confirmation. Biomedicines.

[5] Markovic F., Stjepanovic M., Rancic M., Cekic M., Kontic M. (2025). Real-World Outcomes of First-Line Pembrolizumab Monotherapy in Metastatic NSCLC with High PD-L1 Expression (TPS ≥ 50%): A Multicenter Study from Serbia. Biomedicines.

[6] Kabut J., Stepien G.J., Furgol T., Miciak M., Nafalska N., Stopyra M., Jezierzanski M., Feret K., Gisterek-Grocholska I. (2025). mRNA Vaccines in Modern Immunotherapy for Non-Small Cell Lung Cancer (NSCLC)-A Comprehensive Literature Review with Focus on Current Clinical Trials. Biomedicines.

[7] Pluzanski A., Krzakowski M., Kowalski D., Dziadziuszko R. (2020). Real-world clinical outcomes of first-generation and second-generation epidermal growth factor receptor tyrosine kinase inhibitors in a large cohort of European non-small-cell lung cancer patients. ESMO Open.

[8] Shi K., Wang G., Pei J., Zhang J., Wang J., Ouyang L., Wang Y., Li W. (2022). Emerging strategies to overcome resistance to third-generation EGFR inhibitors. J. Hematol. Oncol..

[9] Reck M., Rodriguez-Abreu D., Robinson A.G., Hui R., Csoszi T., Fulop A., Gottfried M., Peled N., Tafreshi A., Cuffe S. (2016). Pembrolizumab versus Chemotherapy for PD-L1-Positive Non-Small-Cell Lung Cancer. N. Engl. J. Med..

[10] Cortellini A., Tiseo M., Banna G.L., Cappuzzo F., Aerts J., Barbieri F., Giusti R., Bria E., Cortinovis D., Grossi F. (2020). Clinicopathologic correlates of first-line pembrolizumab effectiveness in patients with advanced NSCLC and a PD-L1 expression of ≥50. Cancer Immunol. Immunother..

[11] Descourt R., Greillier L., Perol M., Ricordel C., Auliac J.B., Falchero L., Gervais R., Veillon R., Vieillot S., Guisier F. (2023). First-line single-agent pembrolizumab for PD-L1-positive (tumor proportion score ≥ 50%) advanced non-small cell lung cancer in the real world: Impact in brain metastasis: A national French multicentric cohort (ESCKEYP GFPC study). Cancer Immunol. Immunother..

[12] Piedra A., Martinez-Recio S., Hernandez A., Moran T., Arriola E., Recuero-Borau J., Cobo M., Cordeiro P., Mosquera J., Fernandez M. (2024). First-line pembrolizumab in patients with advanced non-small cell lung cancer and high PD-L1 expression: Real-world data from a Spanish multicenter study. Front. Oncol..

[13] Cortellini A., Friedlaender A., Banna G.L., Porzio G., Bersanelli M., Cappuzzo F., Aerts J., Giusti R., Bria E., Cortinovis D. (2020). Immune-related Adverse Events of Pembrolizumab in a Large Real-world Cohort of Patients with NSCLC with a PD-L1 Expression ≥ 50% and Their Relationship with Clinical Outcomes. Clin. Lung Cancer.

[14] Lee S.M., Schulz C., Prabhash K., Kowalski D., Szczesna A., Han B., Rittmeyer A., Talbot T., Vicente D., Califano R. (2023). First-line atezolizumab monotherapy versus single-agent chemotherapy in patients with non-small-cell lung cancer ineligible for treatment with a platinum-containing regimen (IPSOS): A phase 3, global, multicentre, open-label, randomised controlled study. Lancet.

[15] Wen S.W., Han L., Lv H.L., Xu Y.Z., Li Z.H., Wang M.B., Zhu Y.G., Su P., Tian Z.Q., Zhang Y.F. (2019). A Propensity-Matched Analysis of Outcomes of Patients with Clinical Stage I Non-Small Cell Lung Cancer Treated surgically or with stereotactic radiotherapy: A Meta-Analysis. J. Investig. Surg..

[16] Dautruche A., Filion E., Mathieu D., Bahig H., Roberge D., Lambert L., Vu T., Campeau M.P. (2020). To Biopsy or Not to Biopsy?: A Matched Cohort Analysis of Early-Stage Lung Cancer Treated with Stereotactic Radiation with or Without Histologic Confirmation. Int. J. Radiat. Oncol. Biol. Phys..

[17] Gazdar A.F., Bunn P.A., Minna J.D. (2017). Small-cell lung cancer: What we know, what we need to know and the path forward. Nat. Rev. Cancer.

[18] Landre T., Chouahnia K., Des Guetz G., Duchemann B., Assie J.B., Chouaid C. (2020). First-line immune-checkpoint inhibitor plus chemotherapy versus chemotherapy alone for extensive-stage small-cell lung cancer: A meta-analysis. Ther. Adv. Med. Oncol..

[19] George J., Maas L., Abedpour N., Cartolano M., Kaiser L., Fischer R.N., Scheel A.H., Weber J.P., Hellmich M., Bosco G. (2024). Evolutionary trajectories of small cell lung cancer under therapy. Nature.

[20] Sun J., Nagel R., Zaal E.A., Ugalde A.P., Han R., Proost N., Song J.Y., Pataskar A., Burylo A., Fu H. (2019). SLC1A3 contributes to L-asparaginase resistance in solid tumors. EMBO J..

[21] Lahiri A., Maji A., Potdar P.D., Singh N., Parikh P., Bisht B., Mukherjee A., Paul M.K. (2023). Lung cancer immunotherapy: Progress, pitfalls, and promises. Mol. Cancer.

[22] Kaczmarek M., Poznanska J., Fechner F., Michalska N., Paszkowska S., Napierala A., Mackiewicz A. (2023). Cancer Vaccine Therapeutics: Limitations and Effectiveness—A Literature Review. Cells.

[23] Estape Senti M., Garcia Del Valle L., Schiffelers R.M. (2024). mRNA delivery systems for cancer immunotherapy: Lipid nanoparticles and beyond. Adv. Drug Deliv. Rev..

